# Lupus Vulgaris Plaque Type on the Face Without Pulmonary Involvement: A Rare Case

**DOI:** 10.7759/cureus.51799

**Published:** 2024-01-07

**Authors:** Sankalp Yadav

**Affiliations:** 1 Medicine, Shri Madan Lal Khurana Chest Clinic, New Delhi, IND

**Keywords:** antituberculous drugs, mycobacterium tuberculosis (mtb), extrapulmonary tuberculosis, plaque type, lupus vulgaris

## Abstract

Cutaneous tuberculosis is a rare finding with a difficult diagnosis. This is mainly due to the low sensitivity and specificity of almost all diagnostic tests, accompanied by ambiguity in clinical presentations and non-specific clinical features. A 25-year-old Indian male is presented who reported having a thick, scaly lesion on the left side of his face. A definite diagnosis was achieved after a detailed clinical examination and a detailed diagnostic workup that involved a biopsy. He was put on antituberculous chemotherapy for six months.

## Introduction

Among the earliest diseases known to science, tuberculosis is still a problem in some regions of the world and often brings attention to medical professionals and society at large [1]. Although pulmonary tuberculosis is the most prevalent type, extrapulmonary tuberculosis has also been documented in the published literature [2]. Typically, 8.4-13.7% of all tuberculosis cases are extrapulmonary tuberculosis [3]. Further, extrapulmonary tuberculosis usually manifests in the cutaneous, pleura, abdomen, bone and joints, liver, spleen, lymph nodes, meninges, etc. [2].

Approximately 5.9 cases of cutaneous tuberculosis occur for every 1,000 individuals, making it a rare type of tuberculosis [1]. According to several Indian studies, the total prevalence of cutaneous tuberculosis is 0.25-0.6% [4]. *Mycobacterium bovis*, *Mycobacterium tuberculosis* complex, and occasionally, the Bacillus Calmette-Guérin (BCG) vaccination are the causes of it [5]. Depending on the individual's immune level, the environment, and the type of exposure, it can manifest in a variety of clinical ways [1]. In India, lupus vulgaris is the most prevalent form of skin tuberculosis in adults (75%), whereas scrofuloderma is the most common form in children [1,4]. Lupus vulgaris is a paucibacillary, chronic, progressive, post-primary form of cutaneous tuberculosis that affects people with either a moderate or high level of immunity [1].

Herein, a rare case of a young Indian male, who had a thick, scaly lesion over his left cheek, is presented. The case is unique, as there was no pulmonary involvement or history of contact with tuberculosis, which made the diagnosis challenging in an endemic country.

## Case presentation

A 25-year-old non-diabetic Indian male from a middle-income family presented with a thick, scaly lesion on the left cheek for six months. He was well six months ago when he noted small papules that grew in size and coalesced into the present size. The lesion was painless and not associated with any discharging sinuses. He had no history of fever, weight loss, or any other constitutional sign of tuberculosis. Also, there was no history of skin diseases, immunocompromised illness, immunosuppressive drugs, trauma, tuberculosis, or any contact with a high-risk person. He was a working professional with no history of smoking, alcoholism, or any substance abuse.

**Figure 1:**
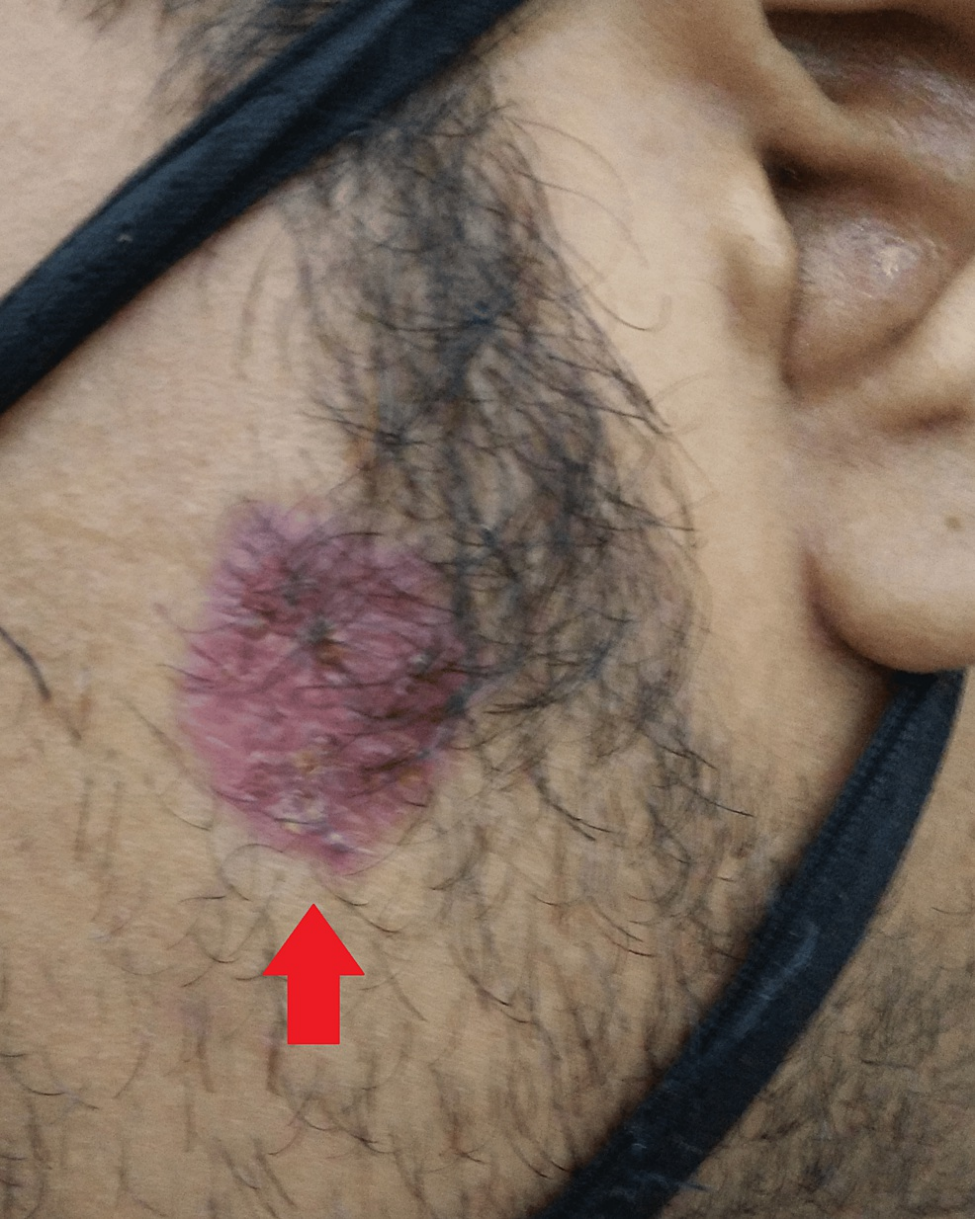
Gross image showing a lesion on the left cheek about 5 cm from the left tragus

A general examination was suggestive of a hemodynamically stable individual. There was no cyanosis, icterus, pallor, lymphadenopathy, edema, clubbing, or koilonychia present. His systemic examination was unremarkable. Local examination showed a single, well-defined, non-tender, pink-colored indurated plaque with central atrophy and a raised periphery with an irregular margin of size 4 × 3 cm and minimal scales over the left side of the cheek. There were no signs of telangiectasia, scarring, adherent scales, follicular clogging, or loss of sensation (Figure 1).

**Figure 2:**
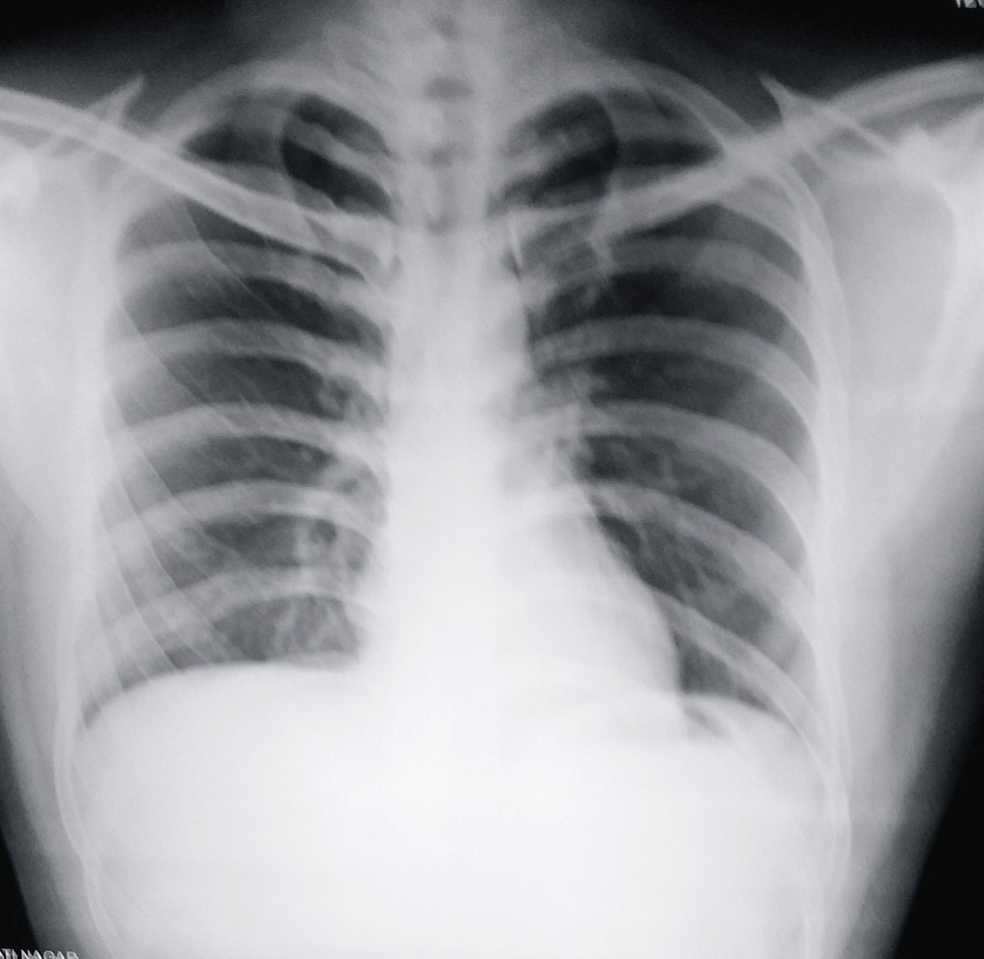
A plain chest radiograph (P-A) view P-A: Posteroanterior

Diascopy showed an apple jelly color at the periphery. Standard biochemical and hematological analyses revealed nothing unusual. After 48 hours, the tuberculin skin test resulted in a strong positive with a 25 mm diameter, but his chest radiograph was normal (Figure 2).

Histopathology of the lesion showed hyperplastic stratified squamous epithelium with hyperkeratosis. The subepithelium showed chronic inflammatory granulation tissue with well-formed epitheloid granuloma with Langhan’s giant cell and stroma showing lymphocytic cell infiltrates. There were no identifiable acid-fast bacilli or characteristic tuberculous follicles. The culture yielded negative results for bacteria, fungus, and acid-fast bacteria. Both the clinical and microscopic characteristics supported the lupus vulgaris diagnosis. The results of the extracutaneous tuberculosis focus screening were negative.

He was initiated on antituberculous drugs for eight weeks with isoniazid, rifampicin, pyrazinamide, and ethambutol in fixed-dose combinations per the national guidelines. For a further four months, the patient received both isoniazid, ethambutol, and rifampicin. A follow-up at the conclusion of the treatment could not be done, as this patient was transferred to a different city; however, his outcome was marked as treatment complete on the national data website, i.e., the Nikshay portal.

## Discussion

Cutaneous tuberculosis is an extremely infrequent occurrence and constitutes about 1-1.5% of the total extrapulmonary tuberculosis [3]. Acute cutaneous miliary, lupus vulgaris, scrofuloderma, tuberculosis verrucosa cutis, orificial tuberculosis, tuberculous gumma, and tuberculous chancre are among the several manifestations of cutaneous tuberculosis [6].

It can be difficult to diagnose cutaneous tuberculosis [7,8]. Either absolute or relative criteria constitute its basis [7]. Identification of *Mycobacterium tuberculosis* from a positive polymerase chain reaction, tissue culture, or guinea pig inoculation result is an absolute criterion [7,8]. However, due to the paucibacillary characteristics of cutaneous tuberculosis, it is a daunting challenge [9]. A comprehensive history and evaluation of lesions, the presence of acid-fast bacillus on lesions, active tuberculosis detected in other organs, the discovery of a tuberculous granuloma on histopathological examination, a positive tuberculin test, and responsiveness to antituberculous drugs are thus additional relative criteria that are available [8].

Lupus vulgaris, or the “wolf” form (given to the lesion as a result of its ulcerating and devouring nature), is a chronic, progressive form of tuberculosis that is rarely recorded [10]. It spreads incessantly from an internal focus, hematogenously or lymphatically, or rarely from external sources (biologic liquids) like infected droplets [9]. People who have become sensitized to *Mycobacterium tuberculosis* often develop cutaneous tuberculosis in the form of lupus vulgaris [8]. Lupus vulgaris manifests in five primary clinical forms: hypertrophy or vegetation, plaque, tumor-like, papular or nodular, and ulcerative. Atrophic and mutilating kinds are among the other clinical forms [1,3].

Most cases of lupus vulgaris present on the face and neck and in young individuals, as seen in the present case [9]. However, when lesions are found in other bodily parts, the situation gets more complicated [3,9]. While facial involvement is widespread in Western countries, buttocks and extremities are usually affected in the Indian population. Re-inoculation is typically the cause of such a pattern, which can be connected to playing barefoot or without shoes [4].

Lupus vulgaris is characterized by an asymptomatic, scaly plaque called a lupoma that forms when reddish-brown papules with a soft texture merge together [3,9]. New papules grow peripherally as the plaque progressively worsens [9]. The lesions have a light brownish-yellow or "apple jelly" tint upon blanching under diascopic pressure [3]. Additionally, a histological investigation will typically reveal characteristic tubercles, either with or without caseation [9]. Dermatoscopy, also called epiluminescence microscopy, is a useful diagnostic tool that compares findings such as finely focused telangiectasias on a yellow-to-golden backdrop to the apple-jelly sign [11]. Furthermore, the polymerase chain reaction's low sensitivity and specificity in lupus vulgaris are a result of the disease's low bacillary burden [9]. The insertion sequence IS6110 has been shown to have a sensitivity that varies, depending on the study, between 70% and 90% and a specificity between 90% and 95% in research laboratories where various genomic segments have been amplified. An extra-dot blot process improves the polymerase chain reaction's sensitivity and specificity [3]. The culture results are typically negative, and only a 6% positivity rate is seen in lupus vulgaris [1].

In a large study by Sellami et al. (2015), out of 88 cutaneous tuberculosis patients, 29 had lupus vulgaris, and of these, 13 had it on the face. They also reported that tuberculin skin test results are usually highly positive in such cases, and the same was seen in the present case [12]. In another study from India, Pai et al. reported plaque-type lupus vulgaris as the commonest; the same type was seen in the present case [4].

Management is essentially medical, with antituberculous drugs in histopathologically confirmed cases. In cases where the diagnosis is challenging, a treatment trial of triple antituberculous therapy may be taken into consideration. Usually, the prognosis is good, and responses are seen earlier (4-6 weeks) as compared to other extrapulmonary tuberculosis [4]. However, untreated cases could end up with severe outcomes [3].

With a reported incidence of 0.5-10.5%, malignant tumors are known to exist in lupus vulgaris, with squamous cell carcinoma being the most prevalent kind [1]. In addition, contractures, tissue damage, and disfigurement are consequences of lupus vulgaris lesions [3].

## Conclusions

A rare case of plaque-type lupus vulgaris is presented, where the diagnosis was achieved by careful clinical examination and histopathology. The case emphasizes the importance of a high degree of suspicion backed by prompt management to avoid fatal outcomes. It is essential that primary care physicians be sensitized about this rare type of tuberculosis, as even in endemic countries, management in such cases is delayed due to ignorance and a lack of training.
